# Climatic Influences on *Cryptoccoccus gattii* Populations, Vancouver Island, Canada, 2002–2004

**DOI:** 10.3201/eid2111.141161

**Published:** 2015-11

**Authors:** Christopher K. Uejio, Sunny Mak, Arie Manangan, George Luber, Karen H. Bartlett

**Affiliations:** Florida State University, Tallahassee, Florida, USA (C.K. Uejio);; British Columbia Centre for Disease Control, Vancouver, British Columbia, Canada (S. Mak);; Centers for Disease Control and Prevention, Atlanta, Georgia, USA (A. Manangan, G. Luber);; The University of British Columbia, Vancouver (K.H. Bartlett)

**Keywords:** climate, Basidiomycota, longitudinal studies, cryptococcosis, temperature, *Cryptococcus gattii*, fungi, climate, Vancouver Island, Canada

## Abstract

Vancouver Island, Canada, reports the world’s highest incidence of *Cryptococcus gattii* infection among humans and animals. To identify key biophysical factors modulating environmental concentrations, we evaluated monthly concentrations of *C. gatti* in air, soil, and trees over a 3-year period. The 2 study datasets were repeatedly measured plots and newly sampled plots. We used hierarchical generalized linear and mixed effect models to determine associations. Climate systematically influenced *C. gattii* concentrations in all environmental media tested; in soil and on trees, concentrations decreased when temperatures were warmer. Wind may be a key process that transferred *C. gattii* from soil into air and onto trees. *C. gattii* results for tree and air samples were more likely to be positive during periods of higher solar radiation. These results improve the understanding of the places and periods with the greatest *C. gattii* colonization. Refined risk projections may help susceptible persons avoid activities that disturb the topsoil during relatively cool summer days.

## Abstract 2

Warmer temperatures are associated with decreased populations in soil and on trees.

Opportunistic fungal infections, such as those caused by *Cryptococcus neoformans*, are common causes of death and illness among persons with compromised immune systems. *C. gattii* is a related fungus that can cause serious illness. Specific genotypes (AFLP4/VGI, AFLP6/VGII) are isolated more commonly from immunocompetent persons, and other genotypes (AFLP5/VGIII, AFLP7/VGIV and AFLP10/VGIV) are isolated more commonly from immunocompromised persons. In 1999, a *C. gattii* genotype that had previously been reported in Brazil and Colombia was first documented on Vancouver Island in the province of British Columbia, Canada (*1*,*2*). The environmental genotypes in British Columbia are primarily VGIIa (AFLP6A, serotype B), VGIIb (AFLP6B, serotype B), and more rarely VGI (AFLP4, serotype B). In 2004, the fungus was identified in the Pacific Northwest region of the United States, and subsequently, *C. gattii* infections have been detected in 8 additional US states (*3*,*4*). Globally, the highest rates of *C.* *gattii* cryptococcosis incidence among humans and animals and the highest rates of positive environmental samples are reported from Vancouver Island (*5*,*6*). The natural habitat of this fungus seems to be a broad range of native trees and the surrounding soil (*2*,*7*,*8*). The epidemiology, nomenclature, historical climate, and population dynamics of *C.* gattii are summarized in the Technical Appendix and Table 1. 

**Table 1:** Summary of findings from longitudinal *Cryptococcus gattii* studies*

Location (reference)	Genotype, serotype	Medium	Highest isolation frequency	Lowest isolation frequency
British Columbia, Canada (*5*)	VGIIa (AFLP6A, serotype B), VG IIb (AFLP6B, serotype B)	Air	Summer: PPT 31 mm/mo, T 11°C–24°C	Winter: PPT 166 mm/mo, T−1°C to 6°C
Bogotá, Colombia (*9*)	B	Tree	Rainy season: RH ≈85%, PPT 120 mm/mo, T 14.4°C–14.8°C	Dry season: Low RH ≈67%, PPT <5 mm, T 14.0°C
Bogotá, Cúcuta, Medellín, Cali, Colombia (*10*)	B	Tree	High RH, low T, low EVAP	Low RH, high T, high EVAP
	C	Tree	Low RH, high T, high EVAP	High RH, low T, low EVAP
Punjab, Haryana, Delhi, Chandigarh, India (*11*)	VGIb (AFLP4)	Tree	Autumn: RH ≈54%, PPT 60 mm/mo, T 25°C; summer: RH ≈30%, PPT 20 mm/mo, T 32°C; rainy: RH ≈60%, PPT 150 mm/mo, T 31°C	Winter: RH ≈55%, PPT 10 mm/mo, T ≈17°C; Spring: RH ≈39%, PPT 11 mm/mo, T 23°C
Jabalpur, India (*12*)	B	Tree	Summer: T 32°C, PPT 0.9–141 mm/mo	Rainy: T 6.6°C–30.6°C, PPT 141–589 mm/mo
S*ã*o Paulo, Brazil (*13*)	B	Tree	November: PPT 244 mm/mo, T 22°C	Other months: PPT 10– 400 mm/mo, T 18°C–26.5°C
Barroso Valley, Australia (*14*)	B	Air	Eucalyptus flowering (Dec–Feb): PPT 0–4.32 mm/mo, T 20.4°C–21.5°C	Other months: PPT 5.08–164 mm/mo, T 8°C–20°C

*Most studies identified the seasons with the greatest or lowest *C. gattii* isolation frequency. Studies commonly examined relative humidity (RH), temperature (T), precipitation (PPT), or evaporation (EVAP).

In general, previous studies examined seasonal versus short-term (e.g., monthly) *C. gattii* associations and primarily focused on *C. gattii* dynamics on trees versus in the air or soil. A limitation of studying seasonal *C. gattii* changes is that it is difficult to disentangle which biophysical conditions (temperature, sunlight, moisture, momentum) most strongly influence *C. gattii* concentrations. For example, which is the primary driver of airborne *C. gattii* levels in southern Australia: temperature, dryness, or both? More frequent *C. gattii* measurements and longitudinal statistics can help distinguish between competing processes. Most long-term studies documented *C. gattii* dynamics on trees; however, seasonality of *C. gattii* may differ in the soil and air (*5*). In particular, airborne *C. gattii* may have the most relevance for human health and deserves further attention. Furthermore, scrutinizing *C. gattii* dynamics in multiple media may provide additional support for conceptualizations of the *C. gattii* life cycle.

Our goal with this study was to determine the relative strength of associations between biophysical conditions and monthly *C. gattii* dynamics from the air, trees, and soil on Vancouver Island, Canada. The first research question examines specific plots from which repeated measurements were made during 2003–2004, and the second question examines only newly sampled *C. gattii* plots during 2002–2004. Based on environmental samples, these investigations were designed to provide insight into the periods with the greatest *C. gattii* area concentrations. This study expands on previous research in the area by studying changes over time, using representative weather stations, considering more biophysical conditions, and using statistics that control for autocorrelation.

## Methods

Concentrations of *C. gattii* in the environmental soil, air, and trees were collected by previously described standardized methods (*5*; Technical Appendix). We evaluated 2 datasets of *C. gattii* VGIIb (AFLP6B, serotype B) previously collected by different sampling strategies: repeatedly measured and newly sampled. The first strategy sporadically resampled a geographic plot after a positive *C. gattii* sample was obtained for this site during 2003–2004. This dataset is similar to the permanently colonized sites analyzed in an ecologic habitat study (*15*). The definition of a plot refers to a specific tree, soil sample 2 meters from the tree base, and the surrounding air. Plots were initially selected with >4 more longitudinal samples. The second strategy analyzed only the first samples from a newly tested plot as analyzed by Kidd et al. (*5*). The sample plots were taken from 9 study areas (Figure). The study areas reported cases in humans, animals, or both or were in biogeoclimatic zones similar to areas with reported cases. Only plots from study areas visited on >3 occasions and from which >1 *C. gattii*–positive sample was obtained were included in the analysis. In each area, new plots were tested in 16%–41% of the study months. Newly sampled plots may reflect *C. gattii* dynamics across the broader study area. 

**Figure:**
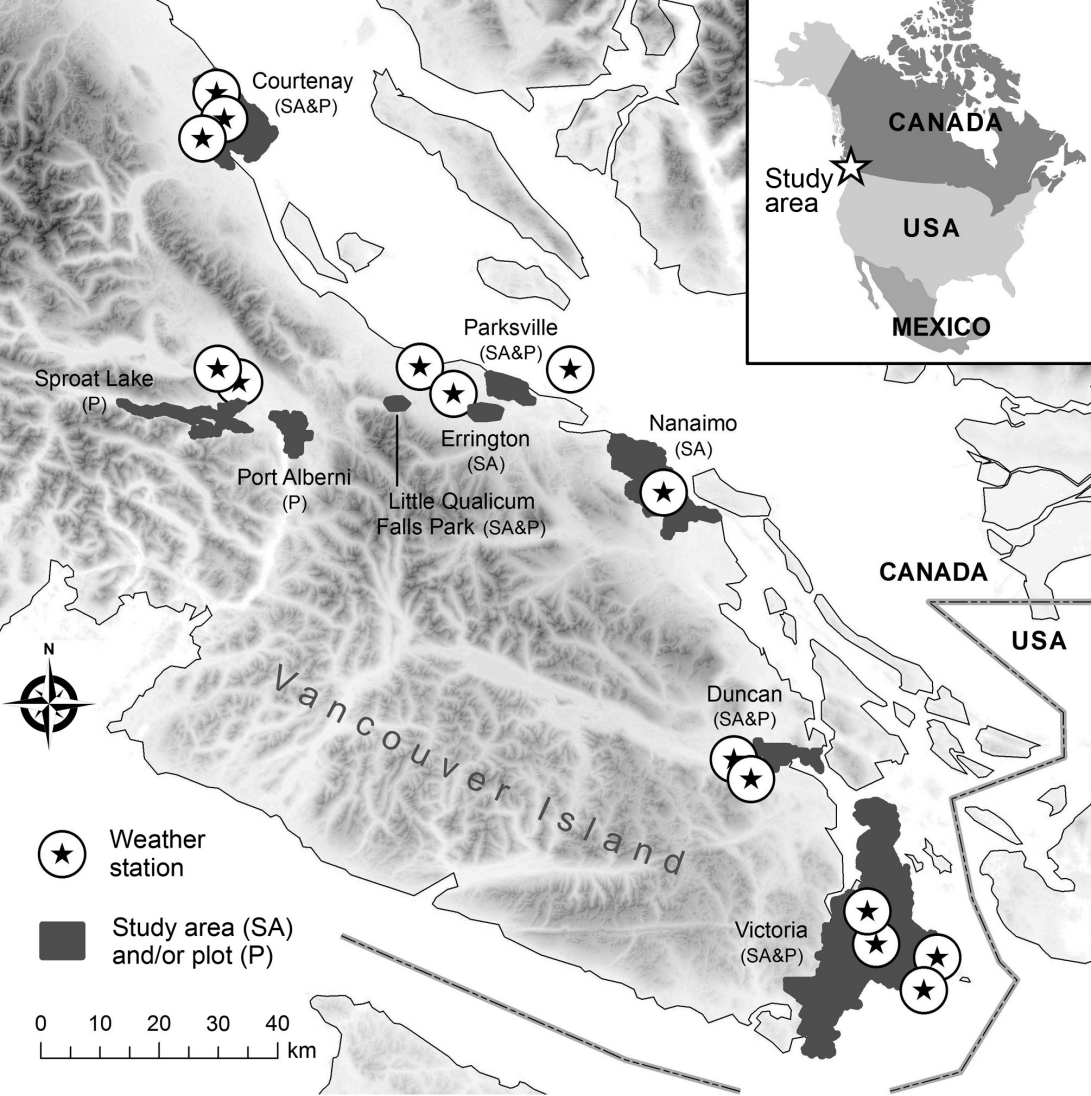
Areas on Vancouver Island, British Columbia, Canada, in which environmental samples were collected to determine *Cryptococcus gattii* concentrations during 2002–2004. Environment Canada provided weather information from 15 stations across the island.

The study examined a broad range of biophysical conditions that plausibly influence population dynamics of fungi in the phylum Basidiomycota. Environment Canada provided daily temperature and precipitation data from 15 weather stations in 7 study areas (http://climate.weatheroffice.gc.ca/Welcome_e.html) (Figure). The second-generation North American Land Data Assimilation System (NLDAS) provided specific humidity, shortwave solar radiation (0.3–3 μm), and wind speed across the domain. Wind speed and solar radiation were infrequently considered in previous studies. Shortwave radiation was converted into Z-scores (number of SDs away from the mean) to align the range of the independent variables and promote statistical convergence. NLDAS uses weather models to interpolate conditions between stations by using physical laws and processes. The spatial resolution of the gridded NLDAS dataset was ≈14 km^2^. Validation shows good agreement between the NLDAS variables used in this study and independent observations (*16*).

There is minimal research to support the selection of periods over which biophysical conditions most strongly influence *C. gattii* dynamics. This analysis broadly considered biophysical conditions over the previous and current day, previous week, and previous month (past 30 days). For each sampling date, *C. gattii* observations for each plot were aligned with the corresponding weather conditions of the surrounding study area.

### Statistical Analyses

Long-term *C. gattii* studies may reanalyze data collected for different purposes, such as surveillance and detection. *C. gattii* was rarely sampled continuously from the same plots. More commonly, repeated measurements were sporadically taken from the same plots. For example, tree A might have been sampled in January–March and August–October, tree B in April–July and November–December, and tree C in April–October. Although no tree was continuously sampled throughout the year, standardizing and pooling the sporadic samples can collectively yield seasonal *C. gattii* information. The analysis maximized the information available from the sporadic samples by use of hierarchical generalized linear and mixed effect models (GLMMs) that control for repeated measurements and clustered sampling (*17*).

GLMMs were used to investigate association of weather conditions with monthly *C. gattii* CFU counts (soil, air) or *C. gattii* presence/absence (trees). Poisson GLMMs with a random effect for each study observation accounted for overdispersion for the soil and air samples. Logistic GLMMs were used to analyze the tree samples. The analysis was conducted in R version 2.15.3 with use of the LME4 package (http://www.R-project.org/). In the first analysis of longitudinal samples, hierarchical random effects controlled for repeated plot measurements and plots nested within study areas.

The random effect in the second analysis accounted for plots nested within study areas. Both analyses controlled for tree genus (cedar, fir, oak, maple, pine, and other). If *C. gattii* were observed <20 times in trees of a given genus, genera were further aggregated into families or lumped into the “other” category. To control for residual spatial autocorrelation, we considered latitude and longitude as candidate independent variables in the analysis. We also controlled for seasonality with fixed-effect indicator variables for winter (November–February), spring (March–May), summer (June–July), and fall (August–October). The GLMM results were reported when the postvariable selection model residuals were not significantly autocorrelated. Residual autocorrelation was tested by autocorrelation and partial autocorrelation functions that were adjusted for missing data.

Intuitively, the *C. gattii* levels for a given month may be strongly related to the previous month’s values. Monthly *C. gattii* samples may exhibit a more complex temporal correlation structure. If the GLMM residuals were significantly autocorrelated, we conducted the analysis on a reduced dataset. For the first analysis, the reduced dataset included plots sampled in sequential months from the plots with >4 longitudinal samples. For the second analysis, the reduced dataset included all first samples in a study area, provided that the study area was sampled in the previous month. Thus, the GLMM controlled for seasonality, plot, or study area–specific random effects, and first-order autoregressive terms for each plot (first analysis) or study area (second analysis). The autoregressive term was the natural logarithm of average *C. gattii* concentration plus 1 (soil, air) or proportion of positive *C. gattii* samples (tree) in the previous month. In the reduced dataset, study areas in which *C. gattii* were observed <20 times were lumped together in the “other area” category.

Because of the relationships among weather conditions, a forward stepwise variable selection procedure involving the Akaike information criterion was used to select the multiple variable models. After a weather variable entered the model, the selection procedure did not consider other temporal aggregations of the same variable. For example, if daily absolute humidity exhibited the most significant *C. gattii* association, weekly or monthly absolute humidity was not tested in the next stepwise iteration. There are minor to moderate differences in the magnitudes of weather conditions across the study area. The statistical results therefore reflect time periods and geographic areas in which weather systematically influences *C. gattii* levels. Weather conditions in the study area were not further standardized to retain the interpretability and biological plausibility of weather conditions for *C. gattii* population dynamics.

## Results

### Plot Level

Table 2 summarizes the mean *C. gattii* concentrations and sample size for the soil and air samples and the proportion of positive tree swab samples. On a plot level (first analysis), weather systematically influenced soil and airborne *C. gattii* levels (Table 3). The soil results from the reduced dataset with plots sampled in sequential months that controlled autocorrelation are reported. The statistical model controlled for a west-to-east gradient of increasing *C. gattii* concentrations across Vancouver Island and for seasonality. Geographic areas and periods with cooler temperatures, lower wind speeds, or both corresponded to the highest *C. gattii* concentrations. Soil concentrations of *C. gattii* were often elevated in the study areas with the coolest temperatures (Parksville and Little Qualicum Falls Park). Average wind speeds were weakest in the study areas surrounding Courtenay and Errington. During October–April, area-averaged (≈14 km^2^) monthly wind speeds were <2 m/s.

**Table 2:** Mean *Cryptococcus* *gattii* concentrations for soil and air samples or proportion of positive tree swab samples, Vancouver Island, British Columbia Canada, 2002–2004*

Medium	Level	Mean *C. gattii* concentration†							
		Parksville	Duncan	Courtenay	Errington	LQFP	Nanaimo	Victoria	Other
Soil, CFU (no. samples)	Plot	2,006 (49)	80,139 (18)	–		–		–	1,635 (28)
Soil, CFU (no. samples)	Area	572 (12)	56 (43)	556 (17)	4 (14)	0 (7)	0 (6)	0 (18)	–
Air, CFU (no. samples)	Plot	100 (113)	202 (38)	2 (34)					
Tree, % (no. samples)	Plot	26 (57)	95 (21)	–		–		60 (15)	50 (22)
Tree, %, (no. samples)	Area	55 (55)	10 (42)	15 (34)		13 (9)	0 (4)	5 (110)	

*LQFP, Little Qualicum Falls Park. †Blank cells indicate areas not included in the analysis. Dashes (–) indicate study areas with a small sample size that were lumped into the column entitled “other.” This information is reported for the plot and area analysis levels.

**Table 3:** Generalized linear and mixed effect model result of the association between weather and *Cryptococcus gattii* in resampled plots in Vancouver Island, British Columbia Canada, 2002–2004

Medium and independent variable	Estimate	SE	95% CI	p value
Soil, CFU*				
Intercept	567.16	167.21	232.7 to 901.5	0.001
Mar−May vs. Nov−Feb	1.06	0.78	−0.50 to 2.626	0.174
Jun–Jul vs. Nov–Feb	15.75	2.38	10.98 to 20.50	<0.001
Aug–Oct vs. Nov−Feb	12.12	1.83	8.45 to 15.78	<0.001
Longitude (°W)	4.47	1.34	1.79 to 7.15	<0.001
Average daily temperature, °C	−1.25	0.19	−1.63 to −0.87	<0.001
Average daily wind speed 1.5−3 m/s	−3.45	0.81	−5.06 to −1.83	<0.001
Average daily wind speed >3 m/s	−5.68	0.99	−7.66 to −3.69	<0.001
Previous month's natural logarithm (*C. gattii* + 1)	0.51	0.11	0.30 to 0.73	<0.001
Garry oak vs. fir/cedar	1.19	1.82	−2.45 to 4.84	0.514
Maple vs. fir/cedar	1.61	1.43	−1.25 to 4.47	0.262

Other tree vs. fir/cedar	−2.82	1.50	−5.82 to 0.18	0.060
Air, CFU†				
Intercept	484.28	94.69	294.8 to 673.6	<0.001
Mar–May vs. Nov−Feb	0.78	1.27	−1.74 to 3.31	0.537
Jun–Jul vs. Nov−Feb	0.89	1.56	−2.23 to 4.01	0.570
Aug–Oct vs. Nov−Feb	2.46	1.05	0.36 to 4.56	0.019
Longitude, °W	3.91	0.76	2.39 to 5.44	<0.001
Daily shortwave solar radiation, watts/m^2^, centered	2.32	0.60	1.11 to 3.52	<0.001
Average daily wind speed 1.5–3 m/s	1.53	0.64	0.24 to 2.82	0.017
Average daily wind speed >3 m/s	−3.97	1.37	−6.71 to −1.21	0.004
Garry oak vs. fir/cedar	0.35	0.84	−1.33 to 2.03	0.680
Maple vs. fir/cedar	−0.27	0.87	−1.99 to 1.47	0.760
Other vs. fir/cedar	0.99	0.73	−0.46 to 2.44	0.174
Swab sample, proportion positive‡				
Intercept	145.29	49.42	46.44 to 244.10	0.003
Mar−May vs. Nov−Feb	2.32	0.80	0.71 to 3.93	0.004
Jun−Jul vs. Nov−Feb	2.22	0.88	0.46 to 3.98	0.012
Aug−Oct vs. Nov−Feb	2.62	0.88	0.87 to 4.37	0.003
Latitude, °N	−2.99	1.01	−5.02 to −0.96	0.003
Proportion of *C. gattii*–positive samples previous month	2.38	0.58	1.22 to 3.53	<0.001
Fir/cedar vs. alder	0.18	0.86	−1.53 to 1.91	0.831
Garry oak vs. alder	−0.23	0.97	−2.17 to 1.72	0.817
Other tree vs. alder	−0.80	1.03	−2.85 to 1.26	0.437

*95 samples, 45 plots, 3 study areas,,Akaike Information Criterion = 648.4. †175 samples, 24 plots, 3 study areas, Akaike Information Criterion = 615.4. ‡115 samples, 44 plots, 4 study areas, Akaike Information Criterion = 117.9.

Airborne *C. gattii* levels for a given month were not associated with those of the previous month. Therefore all plots sampled >4 times were included in the analysis. Similar to the trend for the soil samples, there was an increasing eastward trend of *C. gattii* across the island. Solar radiation intensity was positively associated with airborne *C. gattii* concentrations. The most daily solar radiation is received in the southerly areas (Victoria, Parksville, Duncan) and during May–August. Wind speeds exhibited a more complex, nonlinear relationship to airborne propagules. Moderate daily wind speeds (1.5–3 m/s) may be more likely than less windy days (<1.5 m/s) to entrain *C. gattii* propagules into the air. However, *C. gattii* concentrations were lower on very windy days than on relatively tranquil days. Temperature was not associated with airborne concentrations.

A tree with a positive *C. gattii* sample in a given month was more likely to be positive in the following month. Thus, results are reported from the reduced dataset of trees sampled in sequential months (Table 3). Detection of *C. gattii* in tree samples was not significantly associated with weather conditions**.** Within the study area, northerly regions were less likely to host *C. gattii*–positive trees.

### Study Area Level

Random sampling of new environmental samples during 2002–2004 showed that at the study area level, weather was systematically associated with *C. gattii* in soil and trees (Table 4). Most of the air samples were collected in sequential months, and the small number of air samples from newly sampled plots precluded formal statistical analysis. Consistent with the plot level, concentrations of *C. gattii* in soil were significantly associated with concentrations the previous month. The results of the subset of samples from sequential months are reported. In agreement with the plot-level analyses, higher average temperatures were associated with lower *C. gattii* concentrations in a study area after controlling for seasonality. However, wind speed did not significantly influence concentrations in soil.

**Table 4:** Association between weather and the first *Cryptococcus gattii* sample in study areas, Vancouver Island, British Columbia Canada, 2002–2004*

Medium and independent variable	Estimate	SE	95% CI	p value
Soil†				
Intercept	25.08	15.57	−6.05 to 56.21	0.107
Jun–Juy vs. Mar–May	60.47	25.04	10.39 to 110.50	0.016
Aug–Oct vs. Mar–May	20.24	12.13	−4.02 to 44.49	0.095
Average daily temperature, °C	−4.66	2.15	−8.96 to −0.36	0.030
Cedar vs. alder	1.24	6.56	−11.88 to 14.35	0.850
Fir vs. alder	−2.34	7.44	−17.22 to 12.52	0.753
Oak vs. alder	−1.28	9.49	−20.27 to 17.70	0.893
Maple vs. alder	−0.63	7.07	−14.78 to 13.51	0.929
Other vs. alder	−0.95	7.33	−15.60 to 13.70	0.897
Previous month's natural logarithm(*C. gattii* + 1)	0.65	1.52	−2.38 to 3.69	0.666

Swab sample‡				
Intercept	10.31	2.45	5.42 to 15.19	<0.001
Weekly wind speed, m/s	0.76	0.26	0.24 to 1.28	0.003
Average weekly temperature, °C	−1.23	0.20	−1.63 to −0.82	<0.001
Monthly solar radiation, watts/m^2^, centered	6.25	1.34	3.58 to 8.93	<0.001
Mar−May vs. Nov−Feb	0.92	1.18	−1.43 to 3.27	0.435
Jun−Jul vs. Nov−Feb	2.77	1.70	−0.63 to 6.17	0.103
Aug−Oct vs. Nov−Feb	1.24	1.28	−1.31 to 3.80	0.332
Cedar (western red, yellow) vs. alder	−1.00	1.12	−3.24 to 1.25	0.374
Fir (Douglas, other) vs. alder	−0.55	0.72	−2.00 to 0.89	0.444
Garry Oak vs. alder	0.94	0.90	−0.85 to 2.73	0.296
Maple vs. alder	0	0.85	−1.69 to 1.71	0.997
Other vs. alder	−0.61	0.98	−2.55 to 1.35	0.534
Pine vs. alder	−1.70	1.46	−4.61 to 1.21	0.243
Proportion of *C. gattii*–positive samples previous mo	2.21	0.89	0.43 to 3.98	0.013

*Determined by generalized linear and mixed effect model. †116 samples, 7 study areas, Akaike Information Criterion = 194.7. ‡254 samples, 6 study areas, Akaike Information Criterion = 180.7.

Of note, temperature, wind speed, and solar radiation strongly influenced *C. gattii* dynamics on trees at the study area but not the plot level. Across each study area, a higher proportion of positive tree swab samples from the previous month increased the chances of elucidating *C. gattii* in the current month. The weather relationships were largely consistent with the results from the other media (soil and air). As with the soil samples, geographic areas and periods with warmer temperatures were associated with reduced frequency of *C. gattii* isolation. Similar to the air samples, solar radiation and wind speed were positively associated with frequency of *C. gattii* isolation. *C. gattii* isolation was more likely in southern study areas and during May–August, which had the most solar radiation.

## Discussion

In British Columbia, Canada, *C. gattii* exhibits specialized habitat preferences. It thrives in the area of the Vancouver Island rain shadow (i.e., southeast coast of Vancouver Island and the southwest coast of mainland British Columbia), where winter temperatures are predominantly above freezing and summer temperatures are not too hot (*15*). In the analysis of resampled plots, weather conditions over the previous and current day most strongly influenced *C. gattii* concentrations. For the first *C. gattii* sample analysis, weekly and monthly weather exhibited the best-fitting associations with detection of *C. gattii* in tree swab samples. Granados and Castañeda suggested that conditions up to 15 days before sampling most strongly influence *C. gattii* concentrations (*18*).

Geographic areas and periods with elevated temperatures decreased isolation of *C. gattii* from tree samples and concentration in soil. The results are consistent with *C. gattii* serotype B in Colombia, where *C. gattii* was sampled from the detritus of trees of species with persistent and elevated *C. gattii* concentrations (*Eucalyptus camaldulensis* and *Terminalia cattapa*) (*18*). In that study, the greatest proportions of positive samples were also found during periods of lower temperatures. Similarly, an elevational transect study conducted at elevations of 300–3,000 m found that *C. gattii* concentrations were greater at high elevations with cold temperatures (12°C–18°C annual average temperatures) than in temperate and tropical regions (*19*). In the Vancouver Island study area, average annual temperatures in *C. gattii*–endemic areas were slightly cooler (9.8°C–11.4°C). Outbreaks of *C. gattii* infection in humans or animals in Western Australia, Mediterranean Europe, and North America have been characterized by dry summers or dry winters with warm but not hot monthly temperatures (<22°C) (*20*). Laboratory studies of the optimum growth rates for *C. gattii* and competitors have not been conducted. This knowledge might provide a stronger mechanistic interpretation of temperature associations. According to research of other Basidiomycota, temperature may influence the ecologic niche by regulating the rate of enzyme-catalyzed reactions (*21*).

The aversion of the *C. gattii* strain in British Columbia to higher temperatures may partially account for the difficulty detecting *C. gattii* in environmental samples in warmer neighboring regions. In general, the proportion of *C. gattii*–positive samples declines with increasing southerly distance from Vancouver Island and the Gulf Islands. Prevalence of *C. gattii* in new soil samples (9.6%) and trees (7.7%) on Vancouver Island is remarkably high (*5*). In Washington, USA, British Columbia’s neighbor to the south, *C. gattii* was recovered in 3.0% of air, soil, and tree samples (*5*). This trend continues farther to the south in Oregon, USA, where *C. gattii* was detected in 0–0.6% of tree swab samples (*3*,*22*). The caveat to this trend is that Oregon is host to a different combination of *C. gattii* strains (AFLP6A/VGIIa, AFLP6C/VGIIc) than are British Columbia and Washington (AFLP6A/VGIIa, AFLP6B/VGIIb).

To adapt to biophysical stressors such as temperature, nutrient stress, and radiation, *Cryptococcus* spp. produce melanin. Melanin may increase the integrity of *C. neoformans* cells and make them less susceptible than nonmelanized cells to temperature extremes (*23*). Nutrient stress (glucose and peptone) enhances the production of melanin in *C. gattii* VGI and VGII (*24*). In laboratory *C. neoformans* studies, melanin increases survival to UV-C but not UV-B radiation (*25*,*26*). In our study, periods with more solar radiation (sum of visible, UV, and near-infrared) seem to promote *C. gattii* in the air and trees. Research on *C. gattii* serotype C in Colombia documented a similar association with solar radiation (*18*). To further clarify the role of melanin for mediating environmental stressors, further laboratory studies of *C. gattii* genotypes are needed

The association between windy days and airborne *C. gattii* concentrations may have >1 interpretation. Very windy conditions may be strong enough to transport *C. gattii* away from the local air monitor. It is also possible that these periods coincide with depressed soil *C. gattii* concentrations when there are fewer propagules that can be mobilized. Also, the accuracy of the isokinetic air sampler decreases during periods with stronger wind speeds (*27*).

Collectively, the study results support common conceptualizations of the life cycle of *C. gattii*. Trees and the surrounding soil are permanently colonized and seem to act as *C. gattii* reservoirs. Wind may provide a key process for transferring *C. gattii* from the soil into the air and onto trees in the wider study area. Concentrations of *C. gattii* near the soil surface (0 to <15 cm depth) are greater than concentrations deeper (15–30 cm) in the soil (*3*). Moderate wind speeds may mobilize surface soil and increase local airborne *C. gattii* concentrations. Higher wind speeds may transport *C. gattii* from the soil to trees across the broader area. It is also possible that wind is simply a proxy that coincides with life stages in which propagules are more likely to disperse. *C. gattii* colonization seems to be transitory on many of the recently colonized substrates. *C. gattii* flexibly inhabits and colonizes the soil and specific trees during different seasons, which may decrease intraspecific competition.

The primary route of human *C. gattii* exposure is probably the inhalation of infectious propagules. In the study area, the fungus causes ≈25 clinically diagnosed human illnesses and 4 deaths per year (http://www.bccdc.ca/dis-cond/a-z/_c/CryptococcalDisease/Cryptococcus+gattii.html). According to our results, the highest airborne *C. gattii* concentrations occur during August–October on sunny days with moderately windy conditions. The greatest risk for exposure to *C. gattii* from the soil is during relatively cool June and July summer days. Although these associations are consistent, until more research provides information about the infectious dose for humans, the study results characterize the risk for exposure associated with environmental factors, rather than disease risk. Weather and airborne concentrations of *C. gattii* should be associated with human cryptococcosis incidence; however, onset of documented cryptococcosis cases in British Columbia does not vary by season or month (*28*,*29*). The temporal discrepancy may be masked by the long and variable incubation period of this pathogen. Host factors may be stronger predictors of developing disease risk (*30*). Nonetheless, refined risk projections may benefit susceptible humans and animals living in areas of marginal *C. gattii* transmission.

Technical Appendix Supplemental introduction, methods, and references for study of Climatic Influences on *Cryptoccoccus gattii* Populations, Vancouver Island, Canada, 2002–2004.
