# Correction: Vol. 21, No. 11

**DOI:** 10.3201/eid2204.C12204

**Published:** 2016-04

**Keywords:** errata, erratum, correction

Cryptococcus was misspelled in the title of Climatic Influences on *Cryptococcus gattii* Populations, Vancouver Island, Canada, 2002–2004 (C.K. Uejio al.). The article has been corrected online (http://wwwnc.cdc.gov/eid/article/21/11/14-1161_article).

