# Supplementary material for: Climatic Influences on Cryptoccoccus gattii Populations, Vancouver Island, Canada, 2002–2004
Source: Emerg Infect Dis. 2015 Nov;21(11):1989–96. doi: 10.3201/eid2111.141161 (PMC4622228; doi:10.3201/eid2111.141161)
Supplement: Technical Appendix — Supplemental introduction, methods, and references for study of Climatic Influences on Cryptoccoccus gattii Populations, Vancouver Island, Canada, 2002–2004. [file 14-1161-Techapp-s1.pdf]

Article DOI: http://dx.doi.org/10.3201/eid2111.141161

Climatic Influences on *Cryptoccoccus gattii* Populations, Vancouver Island, Canada

Technical Appendix

Supplemental Introduction

Humans and animals incidentally inhale *C. gattii* propagules and occasionally develop fatal respiratory and nervous systems complications. Individuals, who are older, smoke, take certain medications, or have pre-existing health problems may be more likely to suffer severe complications (*1*). The incubation period between fungal exposure and human case diagnosis is long and variable (range 1.5–36 months) (*2*). Thus, identifying periods with elevated *C. gattii* concentration and potential exposure risk in the environment is difficult to ascertain from disease surveillance of human cases. Environmental samples may provide more information on changes in *C. gattii* area concentrations over time.

Although multilocus sequence typing genetic typing of *C. gattii* strains is now commonplace, classification of *C. gattii* in the literature over the past 30 years includes serotyping (B or C), Restriction Fragment Length Polymorphism analysis (VGI, VGIIa, VGIIb, VGIIc, VGIII, VGIV) and Amplified Fragment Length Polymorphism (AFLP) methods (AFLP4, AFLP5, AFLP6, AFLP7 and AFLP10).

Longitudinal *C. gattii* studies have been conducted in five countries (*3*–*9*). Research groups in Australia, Brazil, Colombia, India, and Canada linked seasonal climatic conditions to *C. gattii* population dynamics. Table 1 in the print article summarizes the physical characteristics of seasons with relatively high and low proportions of positive *C. gattii* samples. The table excludes a small longitudinal study from Northeastern Brazil where *C. gattii* did not clearly exhibit seasonality (*10*). For completeness, temperature and precipitation corresponding to collections in São Paolo, Brazil and the Barroso Valley, Australia were retrospectively added (*8*,*9*,*11*). In general, *C. gattii* exhibits seasonal changes, although the relationships vary by location and *C. gattii* serotype or genotype.

In Southern Australia, Canada, and Colombia (serotype C), *C. gattii* positive samples peaked during the hottest and driest seasons. In Australia, maximum airborne *C. gattii* concentrations temporally coincide with the summertime flowering of *C. gattii's* preferred tree habitat (*Eucalyptus camaldulensis*) (*9*). However, British Columbia peak *C. gattii* airborne concentrations did not overlap with the pollination or flowering of commonly inhabited trees (*3*). The lowest airborne *C. gattii* concentrations were observed during the cooler winter season when precipitation may wash-out fungal propagules. In Colombia, *C. gattii* serotype C was most frequently elucidated from trees in the dry season characterized by high temperatures and solar radiation. However, the more prevalent *C. gattii* serotype B was more likely to be found in the wet season with the opposing biophysical conditions (*5*). The rainy season with moderate temperatures also coincided with *C. gattii* isolation from *Eucalyptus* spp. from a small study in São Paolo, Brazil (*8*). Peak Northern India *C. gattii* VGI (AFLP4) tree isolations coincided with the warmest seasons. However, peak *C. gattii* seasons were more complexly characterized by a wide range of precipitation levels (*6*,*7*). In summary, *C. gattii* populations may peak in the hottest seasons which often, but not necessarily, coincide with dry conditions. For other locations or genotypes, *C. gattii* is more likely to be elucidated in the wet season with moderate temperatures.

Supplemental Data and Methods

Airborne cryptococci were collected using either an Andersen six-stage sieve impaction sampling head (Graseby-Andersen, Atlanta, GA) or a Biotest HYCON RCS centrifugal sampler (Biotest, Frankfurt, Germany). Both samplers were fitted with Niger seed agar (*12*). Media was made in-house using the following formula per liter: *Guizotia abyssinica* seeds (70 g), creatinine (0.78 g), glucose (1.0 g), KH_2PO_4 (1.0 g), chloramphenicol (0.05 g), agar 15 g. *Guizotia* seeds were purchased from a local supplier of bird seed; all other constituents were purchased from Sigma-Aldrich (St. Louis, MO). Air samples were returned to the laboratory for incubation at 30°C and examined daily for evidence of growth of *C. gattii* colonies for up to 10 days (*3*).

Vegetation, primarily mature, native trees, were sampled using commercially available swabs (Starswab, Etobicoke, ON). The sampled trees were Douglas fir (*Pseudotsuga menziesii*, n = 112), Western Red cedar (*Thuja plicata*, n = 24), Garry Oak (*Quercus garryana*, n = 37), Big

Leaf Maple (*Acer macrophyllum*, n = 40), Red Alder (*Alnus rubra*, n = 27), and other (n = 58). Any cold tolerant *Eucalypts* that were found were also sampled, but were never found to be positive. Briefly, swabs were wetted using the supplied Amies transport media (Starswab), then vigorously rubbed around fissures in the bark, or under the bark surface. Swabs were returned to the lab in the Amies transport media, and plated onto Niger Seed agar and incubated similarly to the air sample plates. Based on previous reported work, surface soil samples were collected into plastic, sealable bags and returned to the laboratory (*3*). Approximately 2 g of soil was suspended into 10 mL of sterile water and vortexed. Aliquots of 100 uL were spread onto Niger Seed agar plates, and incubated as previously described. All colonies conforming to the morphology of *Cryptococcus sp.* were isolated and purified. Speciation was confirmed on CGB agar and cryptococcal typing sera (Iatron Laboratories, Tokyo, Japan). Genetic identity was determined using previously described *URA5*-restriction fragment length polymorphism methods (*13*).

Supplemental References

1. MacDougall L, Fyfe M, Romney M, Starr M, Galanis E. Risk factors for *Cryptococcus gattii* infection, British Columbia, Canada. Emerg Infect Dis. 2011;17:193–9. PubMed http://dx.doi.org/10.3201/eid1702.101020

2. MacDougall L, Fyfe M. Emergence of *Cryptococcus gattii* in a novel environment provides clues to its incubation period. J Clin Microbiol. 2006;44:1851–2. PubMed http://dx.doi.org/10.1128/JCM.44.5.1851-1852.2006

3. Kidd SE, Chow Y, Mak S, Bach PJ, Chen H, Hingston AO, et al. Characterization of environmental sources of the human and animal pathogen *Cryptococcus gattii* in British Columbia, Canada, and the Pacific Northwest of the United States. Appl Environ Microbiol. 2007;73:1433–43. PubMed http://dx.doi.org/10.1128/AEM.01330-06

4. Granados DP, Castañeda E. Isolation and characterization of *Cryptococcus neoformans* varieties recovered from natural sources in Bogotá, Colombia, and study of ecological conditions in the area. Microb Ecol. 2005;49:282–90. PubMed http://dx.doi.org/10.1007/s00248-004-0236-y

5. Granados DP, Castañeda E. Influence of climatic conditions on the isolation of members of the *Cryptococcus neoformans* species complex from trees in Colombia from 1992–2004. FEMS Yeast Res. 2006;6:636–44. PubMed http://dx.doi.org/10.1111/j.1567-1364.2006.00090.x

6. Randhawa HS, Kowshik T, Chowdhary A, Prakash A, Khan ZU, Xu J. Seasonal variations in the prevalence of *Cryptococcus neoformans* var. *grubii* and *Cryptococcus gattii* in decayed wood inside trunk hollows of diverse tree species in north-western India: A retrospective study. Med Mycol. 2011;49:320–3. PubMed http://dx.doi.org/10.3109/13693786.2010.516457

7. Bedi NG, Nawange SR, Singh SM, Naidu J, Kavishwar A. Seasonal prevalence of *Cryptococcus neoformans* var. *grubii* and *Cryptococcus gattii* inhabiting E*ucalyptus terreticornis* and *Eucalyptus camaldulensis* trees in Jabalpur City of Madhya Pradesh, Central India. J Mycol Med. 2012;22:341–7. PubMed http://dx.doi.org/10.1016/j.mycmed.2012.09.001

8. Montenegro H, Paula CR. Environmental isolation of *Cryptococcus neoformans* var. *gattii* and *C. neoformans* var. *neoformans* in the city of São Paulo, Brazil. Med Mycol. 2000;38:385–90. PubMed http://dx.doi.org/10.1080/mmy.38.5.385.390

9. Ellis DH, Pfeiffer TJ. Natural habitat of *Cryptococcus neoformans* var. *gattii*. J Clin Microbiol. 1990;28:1642–4. PubMed

10. Lazéra MS, Salmito Cavalcanti MA, Londero AT, Trilles L, Nishikawa MM, Wanke B. Possible primary ecological niche of *Cryptococcus neoformans.* Med Mycol. 2000;38:379–83. PubMed http://dx.doi.org/10.1080/714030957

11. Climate data online [Internet]. Asheville, North Carolina: NOAA National Climatic Data Center; 2014.http://www.ncdc.noaa.gov/cdo-web/.

12. Staib F. Cryptococcocis in AIDS: Mycological diagnostic and epidemiological observations. [AIFO]. AIDS Forsch. 1987;2:363–82.

13. Meyer W, Castaneda A, Jackson S, Huynh M, Castaneda E. IberoAmerican Cryptococcal Study Group. Molecular typing of IberoAmerican *Cryptococcus neoformans* isolates. Emerg Infect Dis. 2003;9:189–95. PubMed http://dx.doi.org/10.3201/eid0902.020246
